# The Role of Plasma Exchange in the Treatment of Refractory Autoimmune Neurological Diseases: a Narrative Review

**DOI:** 10.1007/s11481-021-10004-9

**Published:** 2021-10-02

**Authors:** Saiju Jacob, Gordon Mazibrada, Sarosh R Irani, Anu Jacob, Anna Yudina

**Affiliations:** 1 Institute of Immunology and Immunotherapy, University of Birmingham, Birmingham, United Kingdom; 2 Department of Neurology, Queen Elizabeth Hospital Birmingham, Birmingham, United Kingdom; 3 Oxford Autoimmune Neurology Group, Nuffield Department of Clinical Neurosciences, University of Oxford, Oxford, United Kingdom; 4 Department of Neurology, The Walton Centre NHS Foundation Trust, NMO Service, Liverpool, United Kingdom; 5 Cleveland Clinic Abu Dhabi, Abu Dhabi, United Arab Emirates; 6 TERUMO-BCT Europe, Zaventem, Belgium

**Keywords:** Plasma exchange, Autoimmune Encephalitis, Multiple Sclerosis, Neuromyelitis Optica, Chronic Inflammatory Demyelinating Polyradiculoneuropathy, Myasthenia Gravis

## Abstract

Autoimmune neurological disorders are commonly treated with immunosuppressive therapy. In patients with refractory conditions, standard immunosuppression is often insufficient for complete recovery or to prevent relapses. These patients rely on other treatments to manage their disease. While treatment of refractory cases differs between diseases, intravenous immunoglobulin, plasma exchange (PLEX), and immune-modulating treatments are commonly used. In this review, we focus on five autoimmune neurological disorders that were the themes of the 2018 Midlands Neurological Society meeting on PLEX in refractory neurology: Autoimmune Encephalitis (AE), Multiple Sclerosis (MS), Neuromyelitis Optica Spectrum disorders (NMOSD), Chronic Inflammatory Demyelinating Polyradiculoneuropathy (CIDP) and Myasthenia Gravis (MG). The diagnosis of inflammatory neuropathies is often challenging, and while PLEX can be very effective in refractory autoimmune diseases, its ineffectiveness can be confounded by misdiagnosis. One example is POEMS syndrome (characterized by Polyneuropathy Organomegaly, Endocrinopathy, Myeloma protein, Skin changes), which is often wrongly diagnosed as CIDP; and while CIDP responds well to PLEX, POEMS does not. Accurate diagnosis is therefore essential. Success rates can also differ within ‘one’ disease: e.g. response rates to PLEX are considerably higher in refractory relapsing remitting MS compared to primary or secondary progressive MS. When sufficient efforts are made to correctly pinpoint the diagnosis along with the type and subtype of refractory autoimmune disease, PLEX and other immunotherapies can play a valuable role in the patient management.

**Graphical abstract:**

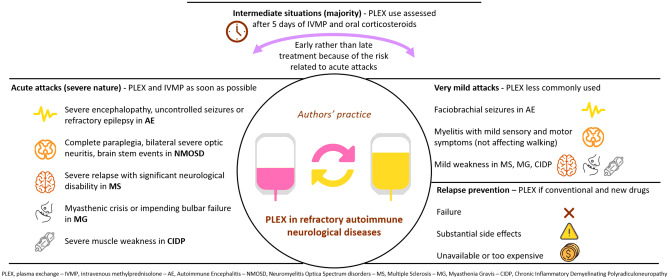

## Introduction

There are numerous autoimmune disorders involving the central nervous system (CNS) and the peripheral nervous system (PNS). They are characterized by abnormal immune responses against antigens expressed within the nervous system. However, the presentation and severity of these diseases can vary greatly (Rubin et al. 2018).

The standard of care for autoimmune neurological disorders in relapse is corticosteroids and immunosuppressive therapy (e.g. azathioprine, mycophenolate) to prevent recurrence of the symptoms. However, a proportion of patients are refractory (i.e. they do not respond to these treatments). Intravenous immunoglobulins (IVIg) and plasma exchange (PLEX) are also widely used to treat neurological disorders, with various degrees of efficacy observed for different disorders (Lehmann and Hartung 2011; Linker and Gold 2008). More recently, immunoadsorption has also emerged as a potential alternative to PLEX for the treatment of neurological disorders. While PLEX consists of fluid replacement with a blood solution such as fresh frozen plasma or albumin, immunoadsorption is a blood purification process through which humoral factors (i.e. disease-specific autoantibodies) can be removed from separated plasma by using a high-affinity adsorbent (Oji and Nomura 2017). The Midlands Neurological Society meeting on PLEX in refractory neurology highlighted five different autoimmune neurological disorders and the available treatment options, in particular the role of PLEX for patients experiencing refractory conditions of the disease. PLEX procedure considerations in these neurological diseases are provided in Table 1.

**Table 1:** Plasma exchange (PLEX) procedure considerations in neurological diseases

**Disease**	**Volume treated**	**Replacement fluid**	**Frequency**
Autoimmune Encephalitis (AE)	1–1.5 PV	Albumin	QOD
Multiple Sclerosis (MS)	1–1.5 PV	Albumin	5–7 plasma exchanges over 14 days
Neuromyelitis Optica Spectrum Disorder (NMOSD)	1–1.5 PV	Albumin	QD or QOD
Chronic Inflammatory Demyelinating Polyradiculoneuropathy (CIDP)	1–1.5 PV	Albumin	2–3x/week until improvement, then taper
Myasthenia Gravis (MG)	1–1.5 PV	Albumin	QD or QOD

PV, plasma volume; QD, every day; QOD, every other day. Adapted from (Schwartz et al. 2016)

In the current review, we focus on the five autoimmune neurological disorders that were presented at the meeting of 2018: Autoimmune Encephalitis (AE), Multiple Sclerosis (MS), Neuromyelitis Optica Spectrum Disorder (NMOSD), Chronic Inflammatory Demyelinating Polyradiculoneuropathy (CIDP) and Myasthenia Gravis (MG). Most of the authors are practicing neuroimmunologists with expertise in specific diseases and aim to provide an overview of the literature and in particular their own perspectives.

## Autoimmune Encephalitis

AE is generally characterized by impaired memory and cognition, often with seizures and/or a movement disorder and sometimes with reduced consciousness or coma, but presentations vary widely across various sub-types (Broadley et al. 2019; Ramanathan et al. 2019). As its laboratory and imaging profiles are frequently normal, AE can be difficult to diagnose.

AE has an incidence of around 0.8/100,000 person-years (Dubey et al. 2018). A small but significant proportion of AE patients is refractory to first- and second-line treatment (Shin et al. 2018), and almost all patients have residual cognitive deficits.

First-line treatment of AE consists of steroids (e.g. methylprednisolone), IVIg and PLEX or immunoadsorption, with corticosteroids being the first choice in most cases. Second-line immunotherapy often consists of rituximab or cyclophosphamide (Shin et al. 2018; Thompson et al. 2018; Titulaer et al. 2013).

While corticosteroids are generally the first choice, treatment with steroids alone is often insufficient to achieve adequate clinical improvements. In these cases, it may be useful to combine steroid treatment with PLEX or IVIg administration to obtain a synergistic effect (Shin et al. 2018). It has been shown that simultaneous PLEX and intravenous methylprednisolone (IVMP) treatment followed transitioning to an IVIg regimen results in improved outcomes on the short term (1–2 months) compared to simultaneous IVIg and IVMP treatment without PLEX (Zhang et al. 2021). PLEX is also thought to positively impact AE by stimulating the proliferation of autoantigen-specific B cells, thereby increasing their susceptibility to immunosuppressants and chemotherapeutic agents (Reeves and Winters 2014; Shin et al. 2018). Response rates of around 65% have been reported for PLEX in refractory AE in a systematic literature review of 120 patients (Suppiej et al. 2016), as well as a retrospective review (DeSena et al. 2015) and a pilot study (Heine et al. 2016) (Table 2). In the latter, the percentage of patients experiencing improvements after PLEX was 83% among those with neuronal cell surface antibodies and 66.7 % among those with antibodies against intracellular-synaptic sites, while none of the patients with intracellular antigens experienced improvements after PLEX (Heine et al. 2016). A recent retrospective study found that 75% of patients with AE respond to PLEX, which also had a favourable safety profile (Moser et al. 2019). Several other studies have also confirmed the efficacy of PLEX in AE (Fassbender et al. 2017). PLEX also seems to be effective in children with AE (Prytuła et al. 2015; Wright et al. 2015).

**Table 2:** Overview of studies evaluating plasma exchange (PLEX)

**Reference**	**Study characteristics**	**N**	**Diagnosis**	**% of response**
**Autoimmune Encephalitis (AE)**				
Heine et al. 2016	Pilot study comparing immunoadsorption and PLEX in autoimmune encephalitis	11	11	67%
DeSena et al. 2015	Retrospective study. Intravenous methylprednisolone versus therapeutic PLEX	14	14	64%
Suppiej et al. 2016	Systematic review.	120	120	64%
Moser et al. 2019	Retrospective study comparing the indication, efficacy and safety of PLEX	12	12	75%
Fassbender et al. 2017	Review of immunoadsorption for AE	98	98	Various
Prytuła et al. 2015	Case series of PLEX in children with acute autoimmune central nervous system disorders	4	4	100%
Wright et al. 2015	Prospective surveillance study of children with N-methyl-D-aspartate receptor antibody-mediated encephalitis	9	9	89% (full recovery)
**Multiple Sclerosis (MS)**				
Correia et al. 2018	Retrospective cohort study. Patients had relapsing remitting MS (85%) or secondary progressive MS (15%)	46	46	80% responded after mean of 7.8 PLEX sessions (41%: complete recovery, 39%: partial recovery)
Stork et al. 2018	Retrospective cohort study evaluating apheresis in patients with three different histopathological patterns of MS	69	69	39% had moderate or marked therapy response within one month of apheresis
Rodriguez et al. 1993	Uncontrolled study of six cases	6	6	100%
Dau 1995	Study in 10 MS patients	10	10	90%
Ruprecht et al. 2004	Case series	10	10	70%
Keegan et al. 2005	Retrospective study	19	19	53%
**Neuromyelitis Optica Spectrum Disorder (NMOSD)**				
Miyamoto and Kusunoki 2009	Report of four patients with NMO who underwent plasmapheresis following intensive intravenous corticosteroid therapy	4	4	100%
Kleiter et al. 2016	Analysis of 871 attacks in 185 patients 142 NMO, 43 NMOSD. PE was chosen as first treatment course for 63 NMO attacks in 27 patients	27 patients 63 NMO attacks	27 patients 63 NMO attacks	30% of attacks: complete remission 51% of attacks: partial remission
Watanabe et al. 2007	Report of six patients with NMO who were unresponsive to high-dose intravenous methylprednisolone	6	6	50%
Abboud et al. 2016	Steroids alone versus steroids plus PLEX for the treatment of acute relapses in NMO	43 patients 65 NMO relapses	43 patients 65 NMO relapses	65%
Bonnan et al. 2018	PLEX initiation delay impact on the outcome of severe NMOSD attacks	60 114 NMO attacks	60 114 NMO attacks	50% for complete improvement if PLEX initiated within 1 day after the attack
**Chronic Inflammatory Demyelinating Polyradiculoneuropathy (CIDP)**				
Mehndiratta et al. 2015	Cochrane literature review, presenting results of two randomized controlled trials	47	47	33–66%
Mahdi-Rogers and Hughes 2014	Cross-sectional study	12	12	42%
Cocito et al. 2010	Retrospective analysis	16	16	56%
Lieker et al. 2017	Prospective study; comparison of PLEX and tryptophan immunoadsorption	9	9	44%
**Myasthenia Gravis (MG)**				
Dau et al. 1977	Case series of five patients	5	5	100% (striking clinical improvement observed in all five patients)
Newsom-Davis et al. 1979	Study evaluating long-term effects of PLEX	7	7	100%
Gajdos et al. 1997	Randomized clinical trial evaluating IVIg and PLEX	41	41	63%
Barth et al. 2011	Comparison of IVIg and PLEX in MG	43	43	57%
Makroo et al. 2008	Retrospective study comparing IVIg and PLEX	19	19	84%
Kumar et al. 2015	Retrospective study	35	35	100%
**Studies evaluating various neurological diseases**				
Weinshenker et al. 1999	Randomized controlled trial evaluating PLEX in acute central nervous system inflammatory demyelinating disease	19*	MS: 6 TM: 2 Marburg: 1 NMO: 2	42% (8/19)
Keegan et al. 2002	Retrospective study	59	MS: 22 ADEM: 10 TM: 6 NMO: 10 Marburg: 7 CIS: 1	44%
Schilling et al. 2006	Retrospective study	16	MS: 14 NMO: 2	70%
Llufriu et al. 2009	Retrospective study	41	MS: 21 CIS: 2 ADEM: 7 NMO: 4 Marburg: 2 TM: 1 ON: 4	63%

ADEM, acute disseminated encephalomyelitis; CIS, clinical isolated syndrome; N, number of patients treated with plasma exchange; Marburg, Marburg's variant of multiple sclerosis; NMO(SD), Neuromyelitis Optica (Spectrum Disorder); ON, optic neuritis; TM, transverse myelitis.

^*^11 patients initially treated with apheresis + 8 treated with apheresis after cross–over

## Multiple Sclerosis

MS is a chronic inflammatory, demyelinating and degenerative disease of the CNS. MS, the most common demyelinating disease, has a prevalence ranging from 2/100,000 population in Eastern Asia and sub-Saharan Africa to more than 100/100,000 inhabitants in North America and Europe (Leray et al. 2016). MS affects >120,000 people in the UK (Mackenzie et al. 2014). The symptomatology of MS varies widely depending on the degree of damage and site of inflammatory, demyelinating and degenerative changes.

The most common type of MS is relapsing remitting MS (RRMS), characterized by relapses and remissions. Incomplete resolution of relapses is associated with accumulation of neurological disability (Lublin et al. 2003). The natural history of RRMS is one of progression, i.e. development of secondary progressive MS (SPMS). If untreated (disease-modifying drugs), the majority of people with RRMS will develop significant neurological disability within 10 years of onset, and 50% will require wheelchair assistance within 20 years. A minority of patients develop primary progressive MS (PPMS), characterized by a gradual progression of neurological disability (Fitzner and Simons 2010).

The standard treatment of MS relapses consists of immunosuppression with corticosteroids, but about 5 to 20% of patients with MS relapses may fail to respond to steroids (Brusaferri and Candelise 2000). The aim of a relapse treatment is to accelerate functional recovery after inflammatory demyelination, alleviate the severity of the relapse and decrease the chance of persistent neurological deficit. Corticosteroids are strong anti-inflammatory agents that exert their actions through various mechanisms including activation of the glucocorticoid receptor and disruption of the mitochondrial membrane potential resulting in apoptosis of T cells, decreasing migration of inflammatory cells into the CNS through decrease expression of adhesion molecules VLA-4 and LFA-1 (Elovaara et al. 1998) and a decrease in intrathecal synthesis of IgG (Sellebjerg et al. 2000).

Patients with steroid-refractory relapses can benefit from PLEX, with reported response rates of 40 to 90% (Stork et al. 2018). The American Academy of Neurology (AAN) (Cortese et al. 2011) guidelines state that PLEX should be considered for the adjunctive treatment of exacerbations in relapsing forms of MS (Level B). PLEX may be considered in the treatment of fulminant CNS demyelinating diseases that fail to respond to high-dose corticosteroid treatment (Level C; the available evidence did not allow to make a recommendation for MS separately). PLEX should not be offered for PPMS or SPMS (Level A) (Cortese et al. 2011). The benefit of PLEX in patients with steroid-refractory relapses is further illustrated by recent studies (Table 2). A Portuguese retrospective cohort study evaluated 46 patients with severe acute relapses of MS, the majority of whom were refractory to corticosteroids. Corticosteroids were used in 94% of cases, without any immediate benefit in 37% and only mild disability recovery in the remaining cases. PLEX was initiated at 33 (±24) days after relapse onset, and 80% of the patients showed recovery after a mean of 7.4 PLEX sessions, with 41% reaching complete recovery (assessed using the Expanded Disability Status Scale [EDSS]) and 39% partial recovery (Correia et al. 2018). 

Another study indicated that the response of steroid-refractive relapses to apheresis may depend on the patient’s histopathological type of disease. The study compared response to apheresis in patients with three histopathologically classified immunological patterns: T cell- and macrophage-associated demyelination (pattern 1), T cell- and macrophage-associated demyelination with immunoglobulin and complement deposits (pattern 2), and oligodendrocyte degeneration (pattern 3). Neurological recovery was observed in five of the 16 patients with pattern 1 disease (31%) and 22 of the 40 patients with pattern 2 disease (55%), but none of the 13 patients with pattern 3 disease exhibited improvement (pattern 2 vs 3 P < .001). When measured by EDSS, the corresponding response rates were 25%, 40% and 0%. Radiological improvements were found in 4 (25%), 22 (56%), and 1 (11%) of patients with patterns 1, 2, and 3, respectively (Stork et al. 2018).

A recent retrospective two-center study compared two types of apheresis: PLEX and immunoadsorption (Lipphardt et al. 2019). Immunoadsorption provides a more selective approach allowing elimination of certain proteins such as antibodies while sparing other plasma proteins (Schroder et al. 2009). The authors concluded that immunoadsorption is equally effective and safe as PLEX in steroid-resistant MS relapses. The highest response rate (74%) to apheresis treatment was observed in patients with RRMS or clinical isolated syndrome (CIS). Interestingly, although the response rate was lower in patients with PPMS/SPMS, 50% of the 22 patients benefited from apheresis (Lipphardt et al. 2019). This observation implies that apheresis could be considered as escalation therapy in progressive MS as well (Lipphardt et al. 2019).

## Neuromyelitis Optica Spectrum Disorder

NMOSD is a CNS disorder that predominantly affects the optic nerves (optic neuritis) and the spinal cord (myelitis), and is mediated by aquaporin-4 immunoglobulin G (IgG) antibodies (AQP4-IgG) in most cases. A proportion of patients with similar presentations have myelin oligodendrocyte glycoprotein IgG antibodies (MOG-IgG), which are also likely pathogenic. NMOSD incidence ranges from 0.053 to 0.4/100,000 person-years (Etemadifar et al. 2015). Currently, both the acute relapses in MOG-IgG disease and AQP4-IgG NMOSD are treated similarly. For the purposes of this review, refractory NMOSD is defined as incomplete or slow recovery from an acute attack, despite corticosteroid treatment. For recurrent attacks, refractory disease is defined as relapses despite treatment with corticosteroids, azathioprine (or mycophenolate) and rituximab.

### PLEX in Acute Attacks

PLEX is recommended in the AAN (2011) guidelines for the treatment of fulminant CNS demyelinating diseases that fail to respond to high-dose corticosteroid treatment. These CNS demyelinating diseases include MS, acute disseminated encephalomyelitis (ADEM), NMOSD and transverse myelitis; the study results did not allow to determine if effectiveness of PLEX varies between the different diseases (Cortese et al. 2011). PLEX is the established second-line therapy in case of steroid resistance in NMOSD in the German and United Kingdom guidelines on treatment of NMOSD (Palace et al. 2012; Trebst et al. 2014).

Reports on the benefit of PLEX are summarized in Table 2. In acute attacks, a response to PLEX has been observed in 35–65% of patients with NMOSD. While PLEX is most effective in the weeks after an acute attack, response has been seen even up to 3 months after onset of the relapse (Abboud et al. 2016; Bonnan et al. 2018). A large retrospective review of 871 relapses treated across Germany supports the escalation from steroids to PLEX, finding that it improved outcomes, particularly in transverse myelitis relapses where PLEX may even be superior to steroids (Kleiter et al. 2016).

A retrospective cohort study focusing specifically on NMO reported that adding PLEX to high-dose IVMP improved the outcome at discharge and on follow-up compared to IVMP alone. Among IVMP + PLEX patients, 65% achieved an EDSS equal or below their baseline at approximately one year follow-up, compared to 35% of the IVMP-only patients (Abboud et al. 2016).

A recent retrospective study comparing PLEX and immunoadsorption, described above for MS, also evaluated 12 patients with NMO. A positive response rate of 67% (8/12 patients) was observed after apheresis treatment. Only two of the 12 patients were treated with immunoadsorption; one of them showed a moderate response while the other improved rapidly after immunoadsorption but lacked sufficient follow-up data (Lipphardt et al. 2019).

When PLEX is ineffective for acute attacks, additional measures that may offer some benefit include IVIg, cyclophosphamide, complement inhibitors (C1 esterase inhibitor [cinryze] and C5 inhibitor [eculizumab]) neutrophil inhibitors (neutrophil elastase inhibitor [sivelestat] and neutrophil migration inhibitor [colchicine]) and eosinophil inhibitors (antihistamines [cetirizine, ketotifen]).

### PLEX for Relapse Prevention

For relapse prevention, until recently, PLEX was used when conventional drugs were exhausted (e.g. azathioprine, mycophenolate, rituximab). The typical regime is two to five exchanges given every four to eight weeks based on response, and used in conjunction with a more conventional agent (e.g. azathioprine + steroids).

A case series of four patients with NMO who underwent PLEX following intensive intravenous corticosteroid therapy reported that all patients showed definite functional improvement after one or several courses of PLEX; two of these patients continued to be treated with intermittent PLEX because of disease refractory to oral agents (Miyamoto and Kusunoki 2009). In a case series of seven patients with NMO refractory to high-dose corticosteroids, an improvement was observed for all patients. In five patients who interrupted their maintenance PLEX therapy, a clinical worsening was observed (Khatri et al. 2012). This finding is currently being further investigated in about 14 patients participating in the “Maintenance Plasma Exchange for Neuromyelitis Optica (MultiPLEX)” prospective observational study (ClinicalTrials.gov: NCT01500681).

It is likely that the three newly approved relapse prevention drugs for NMOSD (eculizumab, satralizumab and inebilizumab) will reduce the need for PLEX.

## Chronic Inflammatory Demyelinating Polyradiculoneuropathy

CIDP is characterized by motor deficits (i.e. numbness and paresthesia followed by weakness), with symptoms gradually worsening over time (Dyck and Tracy 2018). Reported CIDP prevalence per 100,000 population is: 1.61 in Japan (Iijima et al. 2008), 1.90 in New South Wales, Australia (McLeod et al. 1999), 2.84 South East England (Mahdi-Rogers and Hughes 2014), 7.70 Vest-Agder, Norway (Mygland and Monstad 2001).

Standard treatment of CIDP consists of steroids, IVIg, or PLEX. An overview of studies evaluating PLEX to treat CIDP is provided in Table 2.

In a study on patients with chronic inflammatory neuropathies in southeast England, treatment response in patients with CIDP was observed for 68% of the patients treated with corticosteroids, for 63% of those treated with IVIg and for 42% of those treated with PLEX (Mahdi-Rogers and Hughes 2014). Another study reported a response to steroids of 64%, while 78% responded to IVIg and 56% to PLEX. This study noted significant adverse effects of steroid treatment (diabetes, high blood pressure, duodenal ulcer, osteoporosis, psychosis and obesity) and PLEX (difficult access to veins, and deficit of blood coagulation factors) (Cocito et al. 2010).

A 2015 Cochrane systematic literature review (Mehndiratta et al. 2015) concluded that PLEX provides significant short-term improvement in disability, clinical impairment and motor nerve conduction velocity in CIDP, but rapid deterioration may occur afterwards, as based on moderate- to high-quality evidence from two small trials. Adverse events were not uncommon and related to difficulty with venous access, use of citrate and hemodynamic changes (Mehndiratta et al. 2015). AAN (2011) guidelines state that PLEX should be offered as a short-term treatment for patients with CIDP (Level A), while the role of PLEX in the long-term management of CIDP remains to be clarified (Cortese et al. 2011).

A number of small-scale studies indicate that immunoadsorption could constitute a promising and well-tolerated therapeutic alternative for CIDP patients refractory to first-line treatment options, both for short-term and long-term treatment (Dorst et al. 2018; Galldiks et al. 2011). A comparison of tryptophan immunoadsorption with PLEX indicated that immunoadsorption is at least equally effective and safe as PLEX in CIDP patients, with 67% of patients showing clinical improvement after immunoadsorption compared to 44% after PLEX (Lieker et al. 2017).

Importantly, a wrong diagnosis could be the reason for a patient presenting with CIDP that appears resistant to treatment (steroids, IVIg and PLEX). A retrospective study of 59 patients referred with a diagnosis of CIDP found that almost half of these patients (47%) failed to meet minimal CIDP diagnostic requirements (Allen and Lewis 2015). One of the diseases that is often mistaken for CIDP is POEMS, a paraneoplastic syndrome caused by an underlying plasma cell neoplasm. The syndrome is defined by the presence of a peripheral neuropathy (P), a monoclonal plasma cell disorder (M), and other paraneoplastic features, the most common of which include organomegaly (O), endocrinopathy (E), skin changes (S), but they may also have papilledema, edema, effusions, ascites and thrombocytosis. The characteristics of the neuropathy in this syndrome are similar to CIDP, hence explaining the difficulties in diagnosis (Dispenzieri 2005, 2007).

## Myasthenia Gravis

MG is an autoimmune disease characterized by weakness of skeletal muscles and, in over 80% of patients, the presence of autoantibodies to the acetylcholine receptor. Generally, MG occurs more frequently in younger women and older men (Alshekhlee et al. 2009). Estimates of incidence rates vary from three to 30 cases/100,000 person-years (McGrogan et al. 2010).

Treatment of MG consists of cholinesterase inhibitors, steroids and steroid-sparing drugs (e.g. azathioprine, ciclosporin, methotrexate, cyclophosphamide, mycophenolate and rituximab). The AAN (2011) guidelines state that, because of the lack of randomized controlled studies with masked outcomes, there is insufficient evidence to support or refute the efficacy of PLEX in the treatment of myasthenic crisis or MG prethymectomy (Level U for both indications) (Cortese et al. 2011). Similarly, a 2002 Cochrane systematic literature review found no adequate randomized controlled trials. However, the authors did point out that many case series report short-term benefit from PLEX in MG, especially in myasthenic crisis (Gajdos et al. 2002). In the International Consensus Guidance for Management of Myasthenia Gravis, PLEX or IVIg is recommended in combination with high-dose steroids for patients who develop overt MG secondary to immune checkpoint inhibitor treatment (Narayanaswami et al. 2021). The relative rarity of the condition and lack of ambiguity among clinicians (most of whom find PLEX and IVIg to be effective) may be the reason why randomized controlled trials have not been conducted. Reports on the positive impact of PLEX on therapy-refractive MG go back as far as 1977 (Dau et al. 1977) (Table 2).

In current practice, PLEX and IVIg are commonly used to manage myasthenic exacerbations and crises due to their rapid onset of action (Bershad et al. 2008; Sanders et al. 2016). In a comparative study, the two therapies had comparable efficacy and were equally tolerated in adult patients with moderate to severe MG (Barth et al. 2011). IVIg and PLEX are generally not ideal for long-term maintenance therapy due to the short duration of benefits and to the associated side effects (Bershad et al. 2008; Sanders et al. 2016).

About 10–15% of patients with MG are considered to be refractory to standard treatments (Hoffmann and Meisel 2018; Silvestri and Wolfe 2014). Various definitions are being used for refractory MG. Therapy-refractory MG can be defined as chronic courses with moderate to severe symptoms or functional impairment, and is further characterized by ineffective (expanded) standard therapy, repeated myasthenic crises or severe exacerbation, and repeated need for therapy escalation with IVIg, PLEX or immunoadsorption; or in which standard therapy has unacceptable side effects, or there is contraindication for standard therapies due to comorbidities (Hoffmann and Meisel 2018). The Myasthenia Gravis Foundation of America defines refractory MG as “Post-intervention status is unchanged or worse after corticosteroids and at least two other immunosuppressive agents, used in adequate doses for an adequate duration, with persistent symptoms or side effects that limit functioning, as defined by patient and physician” (Sanders et al. 2016).

Escalation strategies recommended for treatment of refractory MG consist of regular IVIg or PLEX, cyclophosphamide and rituximab; other recommendations also include eculizumab, stem cell transplant and newer immunotherapies under trial (Hoffmann and Meisel 2018). As an alternative for PLEX in long-term treatment, immunoadsorption can also be very suitable, as its mechanism of action is more selective (Mantegazza and Antozzi 2018).

## Discussion

In this paper, we have presented an overview of five neurological diseases and the treatment options for patients who are refractory to standard immunosuppression, with a focus on the role of PLEX. Author’s practice is summarized in Table 3.

**Table 3:** Take-home messages (Authors’ practice)

**Acute attacks (severe)**
In the following conditions, PLEX is started as soon as practically possible, along with IVMP (especially in NMOSD and MS relapses):
1. Severe encephalopathy, uncontrolled seizures or refractory epilepsy in AE
2. Complete paraplegia, bilateral severe optic neuritis, brain stem events in NMOSD
3. Severe relapse with significant neurological disability in MS, as seen in spinal and brain stem cerebellar relapses
4. Myasthenic crisis or impending bulbar failure in MG
5. Severe muscle weakness in CIDP
**Acute attacks (mild)**
PLEX is less commonly used in these scenarios:
1. Myelitis with mild sensory and motor symptoms (not affecting walking)
2. Faciobrachial seizures in AE
3. Mild weakness in MG/CIDP/MS
**Acute attacks (moderate)**
For the majority of the situations between these two extremes, PLEX is usually used after assessing the response to five days of IVMP (that is continued by oral corticosteroids) starting usually by the second week of treatment. In general, the authors prefer to treat early rather than late, bearing in mind the high probability of NMOSD and MS attacks leaving behind permanent damage.
**Relapse prevention**
PLEX should be an option when conventional and new drugs fail, side effects are too substantial or when they are unavailable or too expensive.

IVMP, IV Methyl Prednisolone; NMOSD, Neuromyelitis Optica Spectrum Disorder; MS, Multiple Sclerosis; AE, Autoimmune Encephalitis; MG, Myasthenia Gravis; CIDP, Chronic Inflammatory Demyelinating Polyneuropathy

PLEX is a standard first-line therapy in some autoimmune neurological conditions. Following failure or relapse after corticosteroid treatment, PLEX has also shown high response rates in multiple settings. However, the diagnosis of inflammatory neuropathies is often challenging, and ineffectiveness of PLEX can be confounded by misdiagnosis. For instance, POEMS is often wrongly diagnosed as CIDP (Dispenzieri 2005, 2007), and while PLEX is commonly used and effective in the treatment of CIDP, patients with POEMS do not respond well to PLEX (Codron et al. 2017; Dispenzieri 2007).

Even within one disease, not all refractory patients respond to PLEX. This difference may be due to varying immunological aspects in different patients. Additionally, correlations have been uncovered between efficacy of PLEX and different types or patterns within one disease. For example in refractory MS, patients with RRMS respond better to PLEX than patients with SPMS or PPMS (Cortese et al. 2011; Lipphardt et al. 2019).

**Fig. 1:**
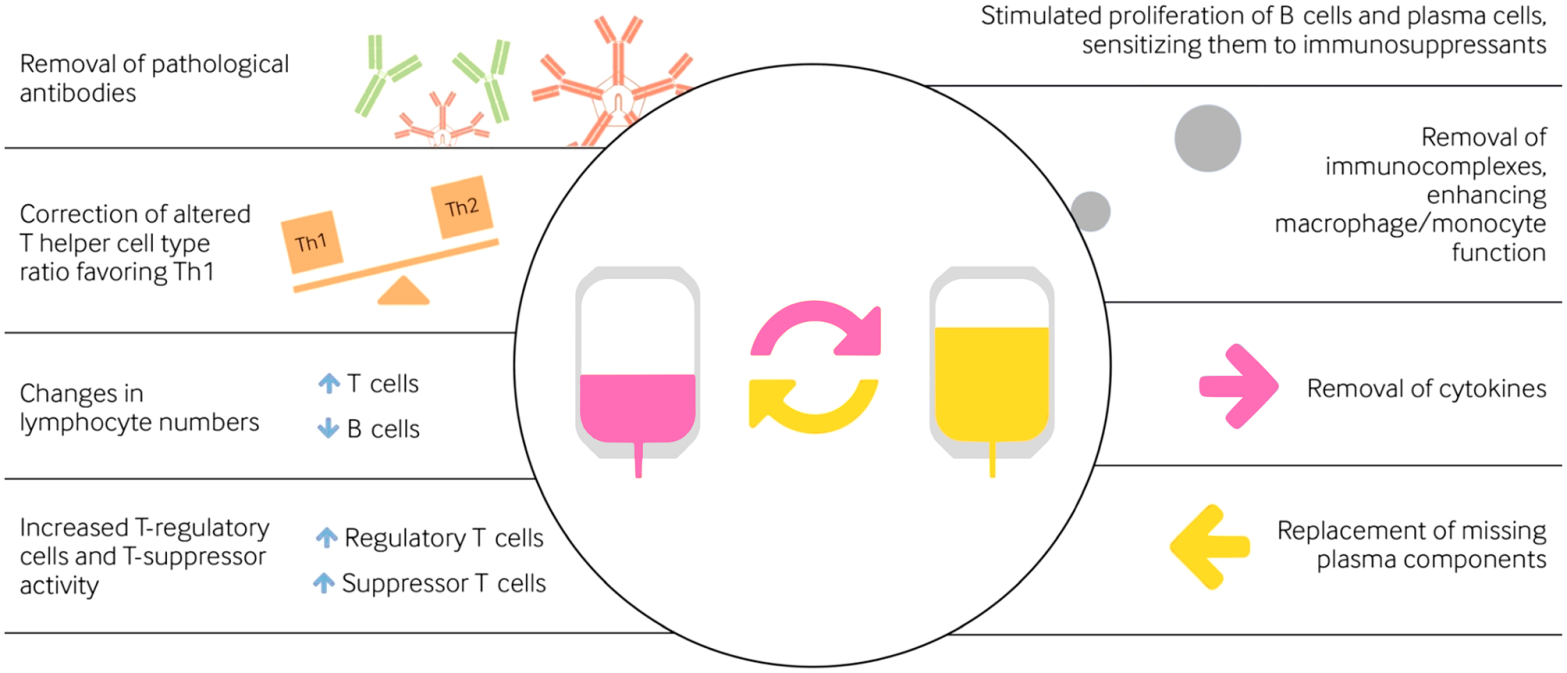
Proposed mechanism of action of plasma exchange (PLEX)

The immunomodulatory aspects of PLEX are not yet fully understood. The most logical method of action in autoimmune diseases is the removal of pathological antibodies. Additionally, PLEX can improve response to other therapies; for instance, by stimulating proliferation of B cells, thereby sensitizing them to immunosuppressants (Reeves and Winters 2014; Shin et al. 2018). Indeed, it has been shown that autoantigen-specific B cells can be observed at high frequencies in the blood of patients with several diseases mediated by autoantibodies, including AQP4, leucine-rich, glioma inactivated 1(LGI1), *N*-methyl D-aspartate receptor (NMDAR) and MOG antibodies (Makuch et al. 2018; Ramberger et al. 2020; Wilson et al. 2018; Winklmeier et al. 2019). Other potential ways in which PLEX can positively impact a disease is through a correction of altered T helper cell (Th) type ratio favoring Th1, changing lymphocyte numbers (more T cells and fewer B cells), increasing T-regulatory cells and T-suppressor activity, removal of immune complexes that enhances macrophage/monocyte function, removal of cytokines, and finally replacement of missing plasma components (Fig. 1, adapted from http://www.pulseline.com.au/community/therapeutic-plasma-exchange) (Reeves and Winters 2014). In autoantibody-mediated diseases, it is likely that these changes have an end-effector function on the autoantigen-specific B cells.

IVIg presents another therapy commonly used to treat refractory neuropathies. It is thought that IVIg and PLEX are similar from a clinical perspective, as for example shown for refractory MG (Barth et al. 2011). However, for certain subsets of patients that are discussed here, PLEX seems to be more beneficial. For example, as concluded in the International Consensus Guidance on the Management of Myasthenia Gravis, PLEX may be more effective than IVIg in MuSK-positive MG and the efficacy of IVIg is less certain in milder MG or ocular MG (Prytuła et al. 2015; Sanders et al. 2016). While anti-AChR antibodies, the most common autoantibodies in MG patients, are predominantly IgG1 and IgG3, anti-MuSK antibodies are predominantly IgG4 (Rivner et al. 2018), which seems to be associated with poor response to IVIg both in MG and CIDP (Wright et al. 2015). IgG4-isotype antibodies do not bind to Ig Fc-receptors and do not activate complement, which may explain the poor response to IVIg when these are involved in the pathogenic mechanism of action (Moser et al. 2019).

One of the perceived advantages of IVIg compared to PLEX is its ease of use, especially in the situation when PLEX is administered via a central line. However, the usage of centrifugal PLEX machines as opposed to the filtration-based ones makes peripheral access possible in over 70% of all cases, and can be as high as over 90% for neurological patients (Bonnan et al. 2018; Fassbender et al. 2017). Peripheral access is minimizing the potential vascular-access-related complications of PLEX and is drastically reducing the rate of catheter-induced infection (Codron et al. 2017; Elovaara and Hietaharju 2010). Peripheral access contributes to the safety and tolerability of PLEX and enables outpatient scenario.

As indicated earlier, delay in PLEX initiation is associated with worsened clinical outcomes. Therefore, having proper planning and priority access for the emergency patients facilitates this treatment option. In the current situation of IVIg shortage, provision of PLEX can help to fill the gap for those patient categories where these two therapies are equally effective (Elovaara and Hietaharju 2010; Kozanoglu et al. 2015; santé 2020).

Finally, both of these treatments have their counter-indications, for example hemodynamic instability, sepsis and hypersensitivity to albumin for PLEX, and renal failure, hypercoagulable states and hypersensitivity to immunoglobulin for IVIg.

Further research will be needed to fully understand the biological mechanisms of PLEX in refractory neurological diseases, to better understand differences in response between the different diseases and subtypes, and to ascertain its place in therapy. Randomized clinical trials provide the highest level of evidence to answer this question. However, such studies are difficult to set up as refractory disease represents only a small subset of already rare conditions.

## Conclusion

The management of the refractory neurological diseases can be challenging. We provide here a fair and balanced review with a focus on the role of PLEX therapy. Further research on the mechanisms of action will help to stratify the patients into groups more likely to benefit from the therapies discussed. Finally, peripheral access and seamless provision are important elements of managing refractory neurology patients with PLEX. 
